# Prospective Surveillance of Invasive Group A Streptococcal Disease, Fiji, 2005–2007

**DOI:** 10.3201/eid1502.080558

**Published:** 2009-02

**Authors:** Andrew C. Steer, Adam Jenney, Joseph Kado, Michael F. Good, Michael Batzloff, Lepani Waqatakirewa, E. Kim Mullholland, Jonathan R. Carapetis

**Affiliations:** University of Melbourne, Melbourne, Victoria, Australia (A.C. Steer, A. Jenney, E.K. Mullholland, J.R. Carapetis); Ministry of Health, Suva, Fiji (J. Kado, L. Waqatakirewa); Queensland Institute of Medical Research, Brisbane, Queensland, Australia (M.F. Good, M. Batzloff); London School of Hygiene and Tropical Medicine, London, UK (E.K. Mullholand); Menzies School of Health Research, Casuarina, Northern Territory, Australia (J.R. Carapetis)

**Keywords:** Streptococcus pyogenes, developing countries, Fiji, molecular epidemiology, streptococcal M protein, research

## Abstract

We undertook a prospective active surveillance study of invasive group A streptococcal (GAS) disease in Fiji over a 23-month period, 2005–2007. We identified 64 cases of invasive GAS disease, which represents an average annualized all-ages incidence of 9.9 cases/100,000 population per year (95% confidence interval [CI] 7.6–12.6). Rates were highest in those >65 years of age and in those <5 years, particularly in infants, for whom the incidence was 44.9/100,000 (95% CI 18.1–92.5). The case-fatality rate was 32% and was associated with increasing age and underlying coexisting disease, including diabetes and renal disease. Fifty-five of the GAS isolates underwent *emm* sequence typing; the types were highly diverse, with 38 different *emm* subtypes and no particular dominant type. Our data support the view that invasive GAS disease is common in developing countries and deserves increased public health attention.

## Abstract 2

These infections are more common and case-fatality rate is higher in Fiji than in industrialized countries.

Invasive disease caused by group A streptococci (GAS) occurs when the bacteria infect a normally sterile site. Invasive GAS disease is often life threatening; mortality rate is ≈10%–15% in industrialized countries, increasing to up to 50% in the presence of streptococcal toxic shock syndrome (*1*,*2*).

A review of the global effects of invasive GAS disease in 2005 estimated that at least 663,000 new cases and 163,000 deaths occur each year (*3*). Although >95% of these cases and deaths occur in developing countries, few data exist about the epidemiology of these infections in developing countries. In addition, few data describe the clinical signs and symptoms, case-fatality rate, and risk factors associated with invasive GAS disease or the molecular epidemiology of invasive GAS disease in developing countries because most published reports originate from industrialized countries (*4*–*8*).

We recently reported incidence of invasive GAS infection in Fiji from a retrospective study in the years 2000–2005 (*9*). This study indicated that potentially substantial effects of invasive GAS disease occur in Fiji. We therefore designed a prospective study with active surveillance to ensure good case ascertainment and the acquisition of more detailed clinical information.

## Methods

This prospective study was performed at the Colonial War Memorial Hospital in Suva, Fiji, during the 23-month period from December 5, 2005, through November 5, 2007. During the first year of the study, we noticed that a considerable number of invasive group C streptococcal (GCS) and group G streptococcal (GGS) infections occurred. We therefore amended the protocol midway through the study (December 5, 2006) to include cases of invasive GCS and GGS infection (year 2). We analyzed 2 datasets: the first was the data available for 23 months of invasive GAS infections, and the second was the data available in year 2 for the 11 months of surveillance of all invasive β-hemolytic streptococcal infections (GAS, GCS, and GGS).

### Setting

Fiji is a nation of ≈330 islands located in the Western Pacific. It has a population of 827,900 persons consisting of 2 major racial groups, indigenous Fijians (57.3%) and Indo-Fijians (37.6%) (*10*). Approximately 49% of the population lives in rural areas (*10*). Of 177 nations on the United Nations Development Programme Human Development Index, Fiji is number 90. Fiji has a gross domestic product per capita of US$6,066, and indexes of wealth are similar for the 2 major racial groups; Indo-Fijians are at a slight disadvantage. The 2002–2003 Household Income and Expenditure Survey estimated that 35.6% of the Indo-Fijian population live in poverty compared with 34.2% of the indigenous Fijian population (*11*,*12*). Life expectancy at birth is 63.8 years for men and 66.8 years for women and is similar for both major racial groups (*11*,*12*). Fiji has an infant mortality rate of 17.2/1,000 population, slightly higher for Indo-Fijians at 19.1/1,000 than for indigenous Fijians at 16.8/1,000 (*11*,*12*). The major hospital, the Colonial War Memorial Hospital (CWMH), is located in the capital, Suva, on the main island of Viti Levu, and primarily serves the Central Division, the largest of the 4 administrative divisions in Fiji. Five subdivisional hospitals are located in the Central Division of Fiji, but most seriously ill patients are admitted to CWMH. This circumstance is due to the 568-bed capacity of CWMH compared with the 81-bed capacity of all 5 subdivisional hospitals combined. CWMH has ≈20,500 admissions per year; the number of indigenous Fijians and Indo-Fijians admitted is proportionate to the population of the Central Division. The total population of the Central Division in 2007 was 340,843: 65% indigenous Fijians and 30.2% Indo-Fijians (*10*). The remainder of the population includes a substantial Chinese population as well as Europeans and Pacific Islanders, including persons from Rotuma, a volcanic island >400 km north of Fiji (it is politically part of Fiji, but its people are ethnically distinct from indigenous Fijians) (*13*).

### Surveillance and Case Definitions

Admission registers at CWMH were checked, and treating physicians were consulted daily for any admitted patient that may have had invasive GAS disease, including those admitted with necrotizing fasciitis, sepsis, and soft tissue infections. The diagnostic microbiology laboratory at CWMH was also contacted daily for any new β-hemolytic streptococcal isolates obtained from sterile and nonsterile sites. We used a consensus case definition of invasive GAS disease developed in 2005 by a World Health Organization/US National Institutes of Health working group; the definition is currently being prepared for publication (F. Rubin, pers. comm.) (Table 1). Cases of streptococcal toxic shock syndrome were defined according to published criteria (*14*). Clinical data were collected from review of the medical records and from information provided by the treating physicians when necessary.

**Table 1:** Case definitions for invasive GAS disease, Fiji, 2005–2007*

Disease	Case definition
Definite	Either of the following: 1. The isolation of GAS from a normally sterile site (e.g., blood, cerebrospinal fluid, or other sterile fluid/tissue). 2. Clinical presentation of necrotizing fasciitis with evidence of GAS infection (e.g., the presence of typical gram-positive cocci on Gram stain or positive streptococcal serology).
Probable	Any of the following: 1. A classic presentation of necrotizing fasciitis without microbiological confirmation. 2. Cellulitis in a patient who is moderately or severely unwell (i.e., unwell and history of parenteral antibiotics and/or admission to hospital) and microbiological confirmation (i.e., group A streptococcal culture of swab or positive streptococcal serology). 3. Other clinically significant infection in a patient who is moderately or severely unwell (i.e., unwell and history of parenteral antibiotics and/or admission to hospital), in conjunction with positive group A streptococcal culture from deep wound swab or biopsy from surgical infection site.

*GAS, group A streptococci.

### Laboratory Methods

All blood cultures collected at CWMH were processed in an automated blood culture machine in the diagnostic microbiology laboratory. Isolates that were suspected of being GAS, GCS, or GGS were subcultured onto sheep blood agar and regrown for Lancefield grouping (Oxoid; Cambridge, UK). Susceptibility testing was performed on sheep blood Mueller-Hinton agar by the disk-diffusion method by using Clnical Laboratory Standards Institute guidelines against a panel of 4 antimicrobial drugs: penicillin (10 µg), erythromycin (15 µg), clindamycin (2 µg), and chloramphenicol (30 µg) (Oxoid). Isolates were transported to the Queensland Institute of Medical Research for *emm* sequence typing according to the standard methods developed by the Centers for Disease Control and Prevention (Atlanta, Georgia, USA) (*15*).

### Statistical Calculations

We used the population of the Central Division from the 2007 national census as the basis for denominator calculations. Because surveillance occurred during 2005 (1 month), 2006 (12 months), and 2007 (10 months), we extrapolated a total population figure for 2005 and 2006 using the pro rata difference between the last official census in 1996 and the latest census in 2007. In calculating average annualized rates, the denominator of person-months was calculated by multiplying each year’s population by the number of months of surveillance for each year (i.e., 2005 population by 1 month, 2006 by 12 months, and 2007 population by 10 months) and adding these totals. The total number of cases over the 23 months was then divided by the total person-months and multiplied by 12 to give average annualized incidence rates with binomial exact 95% confidence intervals (CI). Incidence rate ratios were used to compare rates between ethnic groups. We used χ^2^ calculations of odds ratios (ORs) for univariate analysis of categorical data. Data were analyzed by using Stata version 10.0 (StataCorp, College Station, TX, USA).

### Ethical Approval

Ethical approval was obtained from the Fiji National Research Ethics Review Committee, the Fiji National Health Research Committee, the University of Melbourne Human Research Ethics Committee, and the Queensland Institute of Medical Research Human Research Ethics Committee. We asked all patients for their consent for the collection of more detailed clinical and outcome data as well as for the transport and testing of the clinical isolates. Information sheets in Fijian and English were provided to potential participants before enrollment. We required that all patients provide written informed consent before information was collected. Children were only enrolled if a parent or guardian provided written consent, and we also required written assent from children >10 years of age. When patients did not consent, the case was noted for incidence calculations, but more detailed clinical and outcome data were not collected.

## Results

### Invasive Group A Streptococcal Infections 

#### Epidemiologic Data

Sixty-four cases of invasive GAS disease occurred during the 23 months of surveillance. Sixty-two cases met the criteria for a definite case, and 2 cases met the criteria for a probable case. The average annualized all-ages incidence of invasive GAS disease (both definite and probable) in the Central Division was 9.9 cases/100,000 population/year (95% CI 7.6–2.6). There were an equal number of male and female case-patients. The median age of patients with invasive GAS disease was 51.6 years (interquartile range [IQR] 27.6–66.4 years). The youngest patient was aged 1 month, and 7 patients were <1 year of age, representing an incidence of 44.9/100,000 population (95% CI 18.1–92.5) in this age group (Figure). The peak incidence occurred in patients >65 years (incidence 80.6/100,000, 95% CI 46.1–130.8). Fifty-three cases occurred in indigenous Fijians (incidence 13.1/100,000, 95% CI 9.8–17.1), 5 cases in Indo-Fijians (incidence 2.5/100,000, 95% CI 0.8–5.9), and 6 cases in persons of other races (incidence 12.8/100,000, 95% CI 4.7–27.8). Of the 6 cases in persons of other races, 4 patients were Rotuman (incidence 47.4/100,000, 95% CI 12.9–121.4). When adjusted for the population of the Central Division, the incidence rate ratio for invasive GAS disease in indigenous Fijians versus other races was 2.9 (95% CI 1.5–6.1).

**Figure:**
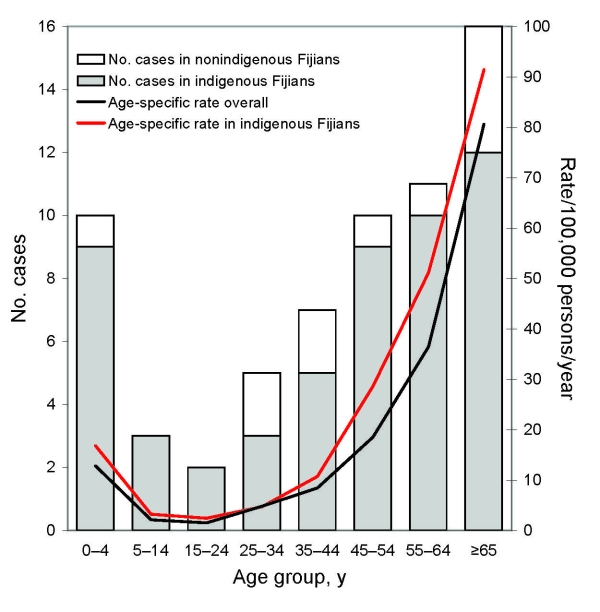
Invasive group A streptococcal disease in the Central Division of Fiji, December 5, 2005–November 5, 2007

#### Clinical Data

Of the 64 case-patients with invasive GAS disease, informed consent to collect clinical and outcome information was obtained from 60. Soft tissue infection (with associated bacteremia) was the most common clinical infection (23 cases [38%]; Table 2); followed by bacteremia with no clinical focus of infection (17 cases [28%]), and necrotizing fasciitis (4 cases [7%]). Three cases (5%) fulfilled the criteria for streptococcal toxic shock syndrome; the clinical focus for these infections was soft tissue infection in 2 case-patients and pneumonia in the third case-patient. Group A streptococci were isolated from blood cultures of 55 (92%) of the 60 case-patients, from both blood culture and other sterile fluid of 4 case-patients, from sterile fluid alone of 3 case-patients, and from nonsterile sites (breast abscess fluid and leg cellulitis swab) of the 2 patients with probable cases. GAS was isolated from blood culture for all 4 case-patients from whom consent to obtain further clinical information was not obtained.

**Table 2:** Clinical signs and symptoms in patients with invasive GAS infection, by age, Fiji, 2005–2007

Clinical signs/symptoms	No. patients by age group			Total no. (%) patients
	0–14 y	15–49 y	>50 y	
Soft tissue infection	2	9	12	23 (38)
Bacteremia with no clinical focus	5	5	7	17 (28)
Septic arthritis	2	1	5	8 (13)
Necrotizing fasciitis	–	2	2	4 (7)
Pneumonia	2	–	2	4 (6)
Gynecologic infection	–	1	1	2 (3)
Osteomyelitis	1	–	–	1 (2)
Peritonitis	–	–	1	1 (2)
Total no. (%) patients	12 (20)	18 (30)	30 (50)	60

*GAS, group A streptococci.

Thirty-eight case-patients (63%) had at least 1 known coexisting chronic medical condition (Table 3). Diabetes was the most common coexisting condition (25 patients [42%]), and was found in 7 of the 12 patients with soft tissue infection who were >50 years of age. No significant difference was found in the rates of underlying disease for indigenous Fijians (62%) compared with rates for persons of other races (70%; p = 0.6). Eighteen patients (30%) had current or recent impetigo. Impetigo was more common in younger patients and was present in 7 of the 10 patients with invasive GAS disease who were <5 years of age and in 4 of the 7 patients <1 year of age. No cases of varicella-zoster virus infection were found in this study, and only 1 patient reported the recent use of a nonsteroidal antiinflammatory drug.

**Table 3:** Underlying coexisting medical conditions in patients with invasive GAS disease, Fiji, 2005–2007

Condition	No. (%) patients
Diabetes	25 (42)
Renal disease	13 (22)
Cardiac disease	13 (22)
Malignancy	5 (8)
Immunosuppression	2 (3)
Lung disease	3 (5)
Liver disease	1 (2)
Any coexisting condition	38 (63)
Only 1 coexisting condition	21 (35)
2 coexisting conditions†	11 (18)
>2 coexisting condition†	6 (10)

*GAS, group A streptococci. †Nine patients had diabetes and renal disease.

#### Outcome and Case-Fatality Rate Data

Of the 60 case-patients for whom outcome data were available, 19 died, representing a case-fatality rate of 32%. The case-fatality rate increased with patient age; peak fatality rate was 44% in those >65 years of age. The odds of death for patients >50 years compared with those of the rest of the patient group was 3.1 (95% CI 0.9–11.7). The youngest patient to die from invasive GAS disease was 1 year and 2 months of age; this was the only death in patients <25 years. No significant difference was found in case-fatality rate for indigenous Fijians (32%) compared with that for persons of other races (30%; p = 0.9). All deaths occurred within 6 days of admission; 5 deaths (26%) occurred within the first 24 hours, 12 deaths (63%) within the first 48 hours, and 15 deaths (79%) within the first 72 hours. The clinical sites of infection in the group of patients that died were similar to the pattern seen overall: 7 patients had a soft tissue infection, 9 patients had bacteremia alone, 2 patients had pneumonia, and 1 had necrotizing fasciitis. All 3 patients with streptococcal toxic shock syndrome died. A coexisting chronic medical condition was associated with an increased risk for death from invasive GAS disease (OR 4.6, 95% CI 1.0–27.7). Twelve patients with diabetes died (OR 3.7, 95% CI 1–13.7), and 8 patients with renal disease died (OR 5.2, 95% CI 1.2–24.2).

Fourteen patients (23%) required surgery as part of their treatment regimen, and 2 of these patients had a leg amputated below the knee. Five patients (8%) were admitted to the intensive care unit, and 1 patient required mechanical ventilation. Twenty-nine patients (48%) were admitted to the hospital for >10 days. Of the 41 survivors of invasive GAS infection, 16 had a residual disability (39%). Overall, 35 patients (58%) died or had a disability as a result of an invasive GAS infection.

#### Laboratory Data

Susceptibility testing was performed on 57 of the 60 isolates, and all 57 isolates were susceptible to all 4 antimicrobial drugs tested. Fifty-five of the 60 isolates were able to be *emm* sequence typed (Table 4); 37 different *emm* types and 38 different *emm* subtypes with no particular dominant types emerged from this series. The most common subtypes were *emm100* and *emm76.4* (4 cases each), followed by *emm11*, *emm33,* and *emm106* (3 cases each). No *emm* type 1 isolates were found. Eight of the 55 isolates *emm* typed were in nonindigenous Fijian patients, and of these, only 1 *emm* subtype was also observed among indigenous Fijian patients.

**Table 4:** *emm* sequence subtypes of 55 invasive GAS isolates and 12 invasive GCS and GGS isolates from hospital surveillance, Fiji, 2005–2007*

GAS *emm* subtype	No. isolates (n = 55)
*emm11†*	3
*emm14.4†*	1
*emm15.1*	1
*emm18.12†*	1
*emm19.4†*	1
*emm22†*	1
*emm33†*	3
*emm52.1*	1
*emm53*	1
*emm57*	2
*emm58*	1
*emm60.1*	1
*emm60.2*	1
*emm63.3*	1
*emm69.1*	1
*emm70*	1
*emm73*	2
*emm75.1†*	1
*emm76.4†*	4
*emm77†*	2
*emm81.3*	1
*emm82.1*	1
*emm86.2*	1
*emm87*	1
*emm100*	4
*emm101†*	1
*emm104*	1
*emm105*	1
*emm106*	3
*emm110*	2
*emm113*	1
*emm116.1*	1
*emm123*	1
*st2037*	1
*st2147*	1
*st6030.1*	1
*st854.1*	2
*stD631*	1
GCS and GGS *emm* subtype	No. isolates (n = 12)
*emm12.8†*	1
*stC36*	2
*stC922*	1
*stG245*	2
*stG643*	1
*stG652*	1
*stG840*	1
*stG5420*	1
*stc74a*	2

*GAS, group A streptococci; GCS, group C streptococci; GGS, group G streptococci. †*emm* subtypes included in the 26-valent vaccine.

### Invasive β-hemolytic Streptococcal Infections after December 5, 2006

#### Epidemiologic Data

In addition to the GAS infections, 4 cases of invasive GCS infection and 14 cases of invasive GGS infection were found in year 2, making a total of 44 cases of invasive β-hemolytic streptococcal infection during the 11 months of surveillance in year 2. This represents an annualized all-ages incidence of 14.1 cases/100,000 population/year (95% CI 10.2–18.9), an incidence rate of invasive GCS disease of 1.3/100,000 (95% CI 0.3–3.3), and an incidence rate of invasive GGS disease of 4.5/100,000 (95% CI 2.5–7.5). Overall, 24 male patients and 20 female patients were infected. The peak incidence was in those >65 years of age (incidence 104.7/100,000, 95% CI 50.2–192.4), and 3 cases occurred in infants. Thirty-four cases occurred in indigenous Fijians (incidence 17.4/100,000, 95% CI 12–24.3), and 10 cases occurred in persons of other races (incidence 8.6/100,000, 95% CI 4.1–15.8); incidence rate ratio for indigenous Fijians was 2 (95% CI 1–4.6).

#### Clinical, Outcome, and Laboratory Data Relating to GCS and GGS Disease

Of the 18 cases of invasive GCS and GGS disease, informed consent to collect clinical and outcome information was obtained for 12 of 14 case-patients with invasive GGS infection and for all 4 case-patients with invasive GCS infection. The clinical spectrum of GCS and GGS infection was similar to that for invasive GAS infection, although the number of patients with bacteremia with no clinical focus was higher, and a case of meningitis in a neonate caused by GCS was added (Table 5). Similar to the experience of patients with invasive GAS disease, a high proportion of patients with invasive GCS and GGS disease had a coexisting chronic medical condition (69%); 44% of patients had cardiac disease, 31% of patients had diabetes, and 6% had renal disease. Six deaths occurred (38%). *emm* sequence typing was performed on 12 isolates (2 GCS and 10 GGS), and 9 different *emm* types were found (Table 4). One was *emm12*, which is normally associated with GAS.

**Table 5:** Clinical signs and symptoms of invasive GCS and GGS infection, Fiji, 2005–2007*

Clinical signs/symptoms	GCS infection	GGS infection	Total
Bacteremia without clinical focus	–	7	7
Soft tissue infection	1	3	4
Endocarditis	1	1	2
Arthritis	–	1	1
Pneumonia	1	–	1
Meningitis	1	–	1
Total	4	12	16

*GCS, group C streptococci; GGS, group G streptococci.

## Discussion

This study confirms the extensive effects that invasive GAS infections have in Fiji and adds to the small amount of existing data indicating that the incidence and case-fatality rates of these infections are ≈3–4 times higher in developing countries than in industrialized countries. A study in Kenya found that the incidence of GAS bacteremia in children <15 years of age was 13/100 000 population and that mortality rate was 25% (*16*), similar to the results in our study. In indigenous populations in wealthy countries, such as Aboriginal Australians and Native Americans, the all-ages incidence of invasive GAS disease is as high as 82.5/100,000 and 46/100,000, respectively (*2*,*17*,*18*).

We observed a nearly 3-fold higher risk for invasive GAS disease in indigenous Fijians. The reasons are not entirely clear and do not appear to be related to socioeconomic status or to coexisting conditions, including diabetes; detailed studies of diabetes in Fiji have shown that the prevalence of diabetes is higher in Indo-Fijians (*19*,*20*) than in indigenous Fijians. One possible explanation is that GAS skin disease is more extensive in indigenous Fijian communities and that this leads to a higher risk for invasive GAS disease. We have observed a higher prevalence in Fiji of bacterial skin disease in indigenous Fijian children (A. Steer, unpub. data), and high rates of GAS skin disease have been found in Pacific Islanders in other parts of the world, including in Maoris and other Polynesians in New Zealand (*21*,*22*).

In most industrialized countries, the annual incidence of invasive GAS disease is 2.5–3.5/100,000 population (*8*,*23*), and the case-fatality rate is 7%–15% (*6*,*8*,*23*–*26*). Although some epidemiologic similarities were found between invasive GAS disease in these countries and in Fiji, such as the high rates for elderly persons with coexisting medical conditions, as well as in the clinical spectrum of disease, major differences stand out. Not only was the all-ages incidence rate higher, but we also found that the incidence rate for children in Fiji was disproportionately high when compared with rates in industrialized countries. For example, the average annualized incidence rate for infants in the United States in 2000–2004 was 5.3/100,000, whereas in Fiji it was 44.9/100,000 (*8*).

The high case-fatality rate in our study (32%) may reflect the late appearance of clinical signs and symptoms, particularly because such a high proportion of deaths occurred in the first 24–48 hours after admission. It may also reflect inadequate treatment of invasive GAS infection, particularly of early sepsis, possibly because of reduced access to intensive care facilities and advanced therapies. The rate of diabetes was also higher in our study than in other studies, potentially further increasing the case-fatality rate.

The *emm* typing profile of invasive GAS isolates was different than that in industrialized countries. In most epidemiologic studies in wealthy countries, a high proportion of disease is caused by a small number of *emm* types that include *emm1* (22% of *emm* types in the United States), *emm3* (9%), and *emm28* (9%) (*8*). None of these *emm* types were found in our study, and we observed a lack of dominant *emm* types. The diversity in *emm* types may reflect the high diversity of GAS in impetigo lesions, as can be seen in tropical settings other than Fiji (*27*).

We observed a substantial number of non-GAS invasive cases in the second year of the study after we expanded our surveillance to include these cases. Individual cases of GCS and GGS invasive disease have been described before (*28*,*29*), but prospective surveillance has not been performed. Our data suggest that surveillance of invasive GAS disease in developing countries should also include surveillance of invasive GCS and GGS disease because the disease profile of these organisms is similar and because control strategies, including vaccines that can offer cross-protection between Lancefield groups, may also be similar.

Identification of factors associated with invasive GAS disease, in particular deaths from invasive GAS disease, can guide disease prevention and disease management efforts. In adults, diabetes was an important risk factor that was associated with death. In children, impetigo appeared to be associated with invasive GAS disease. Control of diabetes in the elderly and control of impetigo in the young may be important disease prevention public health goals. With so many deaths occurring shortly after hospital admission, improved recognition and case management of early sepsis may be important disease management goals. The findings of this study also have implications for GAS vaccine development. Several GAS antigens have been identified as potential vaccine candidates. However, only 1 GAS vaccine, a 26-valent M protein vaccine, has reached phase I and II clinical trials (*30*). Serotypes included in this vaccine were chosen if they were common *emm* types in the United States (*31*,*32*). Although clinical development of the 26-valent vaccine is in its early stages, our results raise concerns about the applicability of this vaccine to developing country settings, because only 10 of 37 *emm* types in our study are types included in the vaccine, accounting for 33% of GAS isolates.

Our study confirms that invasive disease caused by GAS and other β-hemolytic streptococci has substantial effects in Fiji. This study provides the detailed epidemiologic and clinical data on invasive GAS infections from a developing country.
